# Cleavage of TANK-Binding Kinase 1 by HIV-1 Protease Triggers Viral Innate Immune Evasion

**DOI:** 10.3389/fmicb.2021.643407

**Published:** 2021-04-27

**Authors:** Sundararaj Stanleyraj Jeremiah, Kei Miyakawa, Satoko Matsunaga, Mayuko Nishi, Ayumi Kudoh, Akinori Takaoka, Tatsuya Sawasaki, Akihide Ryo

**Affiliations:** ^1^Department of Microbiology, Yokohama City University School of Medicine, Yokohama, Japan; ^2^Division of Signaling in Cancer and Immunology, Institute for Genetic Medicine, Hokkaido University, Sapporo, Japan; ^3^Division of Cell-Free Life Science, Proteo-Science Center, Ehime University, Matsuyama, Japan

**Keywords:** HIV-1 protease, type-I interferon, immune evasion, TANK binding kinase-1, pathogenic fitness

## Abstract

Type-I interferons (IFN-I) are the innate immune system’s principal defense against viral infections. Human immunodeficiency virus-1 (HIV-1) has evolved several ways to suppress or evade the host’s innate immunity in order to survive and replicate to sustain infection. Suppression of IFN-I is one among the multiple escape strategies used by HIV-1 to prevent its clearance. HIV-1 protease which helps in viral maturation has also been observed to cleave host cellular protein kinases. In this study we performed a comprehensive screening of a human kinase library using AlphaScreen assay and identified that TANK binding kinase-1 (TBK1) was cleaved by HIV-1 protease (PR). We demonstrate that PR cleaved TBK1 fails to phosphorylate IFN regulatory factor 3 (IRF3), thereby reducing the IFN-I promoter activity and further reveal that the PR mediated suppression of IFN-I could be counteracted by protease inhibitors (PI) *in vitro*. We have also revealed that mutations of HIV-1 PR that confer drug resistance to PIs reduce the enzyme’s ability to cleave TBK1. The findings of this study unearth a direct link between HIV-1 PR activity and evasion of innate immunity by the virus, the possible physiological relevance of which warrants to be determined.

## Introduction

The innate immune system acts as the broad defense against viral infections (Koyama et al., 2008). Viruses being obligate intracellular organisms, the host cells are equipped with specific antiviral restriction proteins and non-specific pattern recognition receptors (PRRs) to fight these invading pathogens. While the former directly and specifically blocks various stages of viral replication, the latter non-specifically recognizes foreign molecules to trigger the downstream gene induction programs such as the production of interferons (IFN) (Colomer-Lluch et al., 2018). Type-I and III IFNs are produced by a wide variety of cells upon exposure to viruses and provide an instant and brisk antiviral effect to limit the incoming pathogen. Type-II IFN on the other hand is produced by natural killer cells and T-cells and helps in macrophage activation and serves as a link between innate and adaptive immunity (Ferreira et al., 2018; Lee and Ashkar, 2018).

The type-I IFNs are the principal first line of defense against viral infections, and failure to mount an effective IFN response leads to widespread systemic infection in the host (Huang et al., 2019). With respect to the human immunodeficiency virus (HIV), innate immunity is of paramount importance to control HIV-1 infection as it is the principal way not only to prevent widespread establishment of latent reservoirs, but also to deplete them (Soper et al., 2017; Palermo et al., 2019). The success of other strategies to eliminate latent reservoirs for a functional cure could be enhanced if the innate immunity against HIV-1 is strengthened (Altfeld and Gale, 2015).

Type-I and III IFNs are produced through one or more intracellular signaling pathways involving several proteins, which are initiated by the sensing of pathogen-associated molecular patterns (PAMPs) by the PRRs. PAMP recognition by PRRs leads to recruitment of various intracellular adaptor proteins to form a signaling complex which orchestrates the downstream events leading to IFN production. Despite the diverse types of PRRs providing the upstream sensing signals, the TRAF Family Member Associated NFKB Activator protein binding kinase-1 (TBK1) is found to be invariably involved in the signaling complexes of almost all the type-I and III IFN induction pathways currently identified (Beachboard and Horner, 2016; Ni et al., 2018). The key event is the autophosphorylation of TBK1 in the signaling complex. Phosphorylated TBK1 in turn phosphorylates IFN regulatory factor 3 (IRF3) which undergoes dimerization, detaches from the signaling complex and translocates to the nucleus to bind with specific promoters inducing type-I and III IFN production (Hu et al., 2018).

Viruses have evolved along with their hosts and possess mechanisms to overcome or evade innate immunity in order to propagate within the host (Domingo-Calap et al., 2019). Viruses lacking this ability could not infect and replicate inside the host (Iyer et al., 2017). The human immunodeficiency virus (HIV) has developed several ways to thwart the defenses posed by the innate immune system (Yin et al., 2020). These could be broadly classified into two; counteraction of host restriction factors and attenuation of IFN response. The accessory proteins of HIV have shown to counteract the effects of various cellular anti-retroviral restriction factors such as APOBEC3G, SAMHD1, TRIM5α, tetherin and others (Colomer-Lluch et al., 2018). The strategies employed by HIV-1 to dampen the IFN response can be further classified into HIV-1 protease (PR) mediated or HIV-1 PR independent mechanisms. The salient PR independent mechanisms include; evading cytosolic PRR sensing by hijacking host factors to mask its PAMPs (Ringeard et al., 2019), reducing their time of stay in the cytoplasm to minimize the chances of PRR detection (Dharan et al., 2017) and deploying one or more viral accessory proteins to attenuate the functions of cellular molecules involved in signaling pathways of IFN production (Beachboard and Horner, 2016).

Although HIV-1 PR was initially thought to only participate in maturation of viral progeny with their intra viral action in extracellular viruses, recent findings have revealed several mechanisms by which HIV-1 PR interferes with host cell proteins to suppress IFN production and innate immunity (Rajput et al., 2011; Wagner et al., 2015). In the present study, we identify yet another novel, PR dependent immune evasion mechanism used by HIV-1 to suppress IFN production. We demonstrate the HIV-1 PR-mediated proteolytic cleavage of TBK1, the key signaling molecule of type I and III IFN production pathways. We also confirm this finding by demonstrating the inability of the cleaved TBK1 to activate IRF3, thereby diminishing the downstream signaling events of IFN induction pathway and the reversal of this effect by protease inhibitors (PI). Furthermore, we show that drug resistance mutations of HIV-1 PR to PIs reduce the TBK1 cleaving ability of HIV-1 PR which could probably reflect the loss in the pathological fitness with evolution.

## Materials and Methods

### Cell Lines and Transfection

HEK293 cells from ATCC were cultured in DMEM supplemented with 10% (V/V) fetal bovine serum (FBS) and 1% penicillin-streptomycin (PS) in a 5% CO_2_ at 37°C. To generate T7 cells (THP-1 cells encoding HIV-1 provirus), cells were infected with VSV-G-pseudotyped HIV-1(ΔEnv)-Luc at a multiplicity of infection (MOI) of 0.5 and selected by limiting dilution. T7 cells showed low expression of luciferase in the steady state, but luciferase activity increased when TNF-α was added, suggesting that the viral genome was latent. THP1 and T7 cells were grown in RPMI containing 10% FBS and 1% PS in 5% CO_2_ at 37°C. Cell transfection was performed using lipofectamine3000 (Thermo), according to the manufacturer’s instructions.

### Plasmids

Human kinase library is listed in Supplementary Table 1 with sequence data of each kinase obtained from HUGO gene nomenclature committee (HNGC), accessible at https://www.genenames.org/. TBK1 cDNA was subcloned into pCMV-HA vector (Clontech) or pEU (CellFree Sciences). TBK1 mutants were generated by PrimeSTAR mutagenesis basal kit (Takara). Plasmids encoding HIV-1 Gag and Gag-Pol were described previously (Kudoh et al., 2014). FLAG-p6^∗^PR was synthesized as explained in previous study (Chiu et al., 2006). pGL3-based IFNβ-promoter plasmid was obtained from addgene (#102597). HIV-1 drug resistant mutants were contributed by Dr. Masashi Tatsumi.

### Wheat Germ Cell Free Protein Production

*In vitro* transcription and cell-free protein synthesis was performed as described previously (Sawasaki et al., 2005). Transcripts were made from each of the DNA templates mentioned above using SP6 RNA polymerase. The synthetic mRNAs were then precipitated with ethanol, collected by centrifugation and washed. Each mRNA (typically 30–35 μg) was added to the translation mixture and the translation reaction was performed in the bilayer mode with slight modifications (Sawasaki et al., 2002). The translation mixture that formed the bottom layer consisted of 60 A260 units of wheat germ extract (CellFree Sciences) and 2 μg creatine kinase (Roche Diagnostics K. K., Tokyo, Japan) in 25 μl SUB-AMIX solution (CellFree Sciences). SUB-AMIX contained (final concentrations) 30 mM Hepes/KOH at pH 8.0, 1.2 mM ATP, 0.25 mM GTP, 16 mM creatine phosphate, 4 mM DTT, 0.4 mM spermidine, 0.3 mM each of the 20 amino acids, 2.7 mM magnesium acetate, and 100 mM potassium acetate. SUB-AMIX (125 μl) was placed on the top of the translation mixture, forming the upper layer. After incubation at 16°C for 16 h, protein synthesis was confirmed by SDS-PAGE. For biotin labeling, 1 μl (50 ng) of crude biotin ligase (BirA) produced by the wheat germ cell-free expression system was added to the bottom layer, and 0.5 μM (final concentration) of D-biotin (Nacalai Tesque, Inc., Kyoto, Japan) was added to both upper and bottom layers, as described previously (Matsuoka et al., 2010).

### AlphaScreen Assay

*In vitro* cleavage activity assays of HIV-1 PR were carried out in a total volume of 15 μl consisting of 100 mM Tris–HCl pH 8.0, 0.01% Tween-20, 1 mg/ml BSA, 1 μl crude recombinant protease (∼ 0.75 μM) and 0.5 μl crude recombinant FLAG-biotin-tagged CA/NC (∼ 0.037 μM) at 37°C for 1 h in a 384-well Optiplate (PerkinElmer, Boston, MA, United States). To assay the effects of HIV-1 PR on various human protein kinases, 3 μl HIV-1 PR and human PK each was incubated at 37°C for 10 min, FLAG-biotin-tagged CA/NC or GST-biotin-tagged p2–p7 was added and the reaction further incubated at 37°C for 1 h in a 384-well Optiplate. In accordance with the AlphaScreen IgG (Protein A) detection kit (PerkinElmer) instruction manual, 10 μl of detection mixture containing 100 mM Tris–HCl pH 8.0, 0.01% Tween-20, 1 mg/ml BSA, 5 μg/ml Anti-FLAG antibody (Sigma-Aldrich, St. Louis, MO, United States) or Anti-GST antibody (GE Healthcare, Buckinghamshire, United Kingdom), 0.1 μl streptavidin-coated donor beads and 0.1 μl anti-IgG (Protein A) acceptor beads were added to each well followed by incubation at 26°C for 1 h. Luminescence was analyzed by the AlphaScreen detection program. Each assay was performed in triplicate, and the data represent the means and standard deviations of three independent experiments.

### Luciferase Assay

HEK293 cells in 12-well plates transfected with plasmids encoding IFNβ-promoter-Luc (100 ng), HA-TBK1 (100–400 ng), and p6^∗^PR (100 ng) were allowed for 48 h of incubation and were then lysed and added with equivalent volume of Bright-Glo Substrate (Promega). Luciferase activity was measured with GloMax Discover System (Promega).

### Immunoblotting and Protein Sequencing

For recombinant protein analysis, 3 μl crude recombinant viral protease (∼ 0.75 μM) and 7 μl crude FLAG-biotin-tagged recombinant proteins were incubated at 37°C for 2 h. To assay the effect of HIV protease inhibitors, 3 μl crude recombinant HIV-1 protease and 1 μl of 10 μM protease inhibitor amprenavir (Sigma-Aldrich) were incubated at 37°C for 10 min followed by addition of 6 μl crude FLAG-biotin-tagged recombinant proteins and incubated at 37°C for 120 min. Proteins were separated by SDS-PAGE and transferred to a PVDF membrane (Millipore) according to standard procedures. Immunoblot analysis was carried out with anti-FLAG (M2) antibodies (Sigma-Aldrich) or Streptavidin-HRP conjugate (GE Healthcare) according to the procedure described above. For fluorescent imaging, immunoblotted proteins were detected by Alexa592-anti-mouse antibodies (N-cleaved fragments), and Alexa488-streptavidin (C-cleaved fragments). The labeled proteins were visualized using a Typhoon Imager (GE Healthcare). To determine the cleavage site in TBK1 by HIV-1 PR, the cleaved fragments on the PVDF membrane were extracted and washed with methanol and were outsourced to commercial laboratory for sequencing.

For intracellular protein analysis, cells expressing GagPol and TBK1 were harvested with immunoblotting sample buffer. In Figure 2B, cells were treated with APV (0.02–2 μM) 16 h before harvesting. After SDS-PAGE and subsequent membrane transfer, the membranes were probed with primary antibodies and horseradish peroxidase-conjugated secondary antibodies (GE Healthcare). The primary antibodies used were as follows: anti-HA (Roche), FLAG (Sigma-Aldrich), Gag p24 (NIH AIDS Reagent Program), IRF3, IRF3(pS396), TBK1(pS172) (Cell Signaling Technology), and vinculin (Sigma-Aldrich). Detected proteins were visualized using a FluorChem digital imaging system (Alpha Innotech). Band analysis was performed with ImageJ software (National Institutes of Health).

### Immunofluorescence Analysis

Cells were fixed with 4% paraformaldehyde and permeabilized with 0.5% Triton X-100, and then were blocked with Blocking One (Nacalai) at room temperature for 15 min. The cells were incubated with antibodies against HIV-1 p24 antigen (NIH AIDS Reagent Program) and IRF3 (Cell Signaling Technology) at room temperature for 1 h. After incubation, cells were stained with Alexa 568 or 488-labeled anti-IgG antibody (Thermo) for 1 h at room temperature. The nucleus was stained with ProLong Gold Antifade Mountant with DAPI (Thermo). The images were taken by fluorescence microscope BZ-9000 (Keyence).

### Protease vs. TBK1 in Infection Model

The HIV-1 latently infected T7 or the uninfected THP1 monocytic cell lines were transfected with 10 ng/ml poly(I:C) (Sigma-Aldrich) in 12 well plates using lipofectamine3000 (Thermo), according to the manufacturer’s instructions (Ranganath et al., 2016). Bryostatin-1 (Sigma-Aldrich) at a final concentration of 25 ng/ml was included in the medium of T7 cells immediately after poly(I:C) transfection, followed by the addition of 10 μM of the protease inhibitor indinavir (Sigma-Aldrich) in respective wells. The cells were incubated in 5% CO_2_ at 37°C. After 48 h of incubation, mRNA was extracted from cells using RNeasy mini kit (QIAGEN). The amount and purity of the mRNA was assessed using NanoDrop One (Thermo Scientific) and stored at −80±C until further use. Reverse transcription was performed using ReverTra Ace qPCR-RT kit (TOYOBO). PCR reagents were purchased from Takara and the primers for IFNβ1 and ISG15 were obtained from Eurofins with the following sequences; IFNβ1 Fwd: GTCAGAGTGGAAATCCTAAG and IFNβ1 Rev: ACAGCATCTGCTGGTTGAAG (Hu et al., 2010) and ISG15 Fwd: CTCTGAGCATCCTGGTGAGGAA and ISG15 Rev: AAGGTCAGCCAGAACAGGTCGT (Matsunaga et al., 2020). The expression of genes was quantified using CFX96^TM^ Real-time system (Bio-Rad) and normalized against the expression of β-actin in the corresponding cells.

### Statistical Analysis

All bar graphs present means and standard deviation obtained from three replicates. The statistical significance of differences between two groups was evaluated by two-tailed unpaired *t*-test. A *p* value of <0.05 was considered statistically significant.

## Results

### Screening for Human Kinases Cleaved by HIV-1 Protease

HIV-1 PR is an essential enzyme required for the virus to complete its life cycle. PR gets packed into the immature virions that bud out of the infected cells and cleave the viral precursor proteins into their functional form thereby generating mature extracellular infectious viruses. However, apart from their action within the viral particle, HIV-1 PR has been shown to influence cellular functions by interacting with various host proteins in the infected cell (Yang et al., 2012). Protein kinases (PK) are enzymes which are involved in carrying out biological functions of the cell by phosphorylating proteins. The interaction of HIV-1 PR with host cellular kinases both in the cytoplasm and the nucleus have been reported previously (Devroe et al., 2005; Wagner et al., 2015).

**FIGURE 1:**
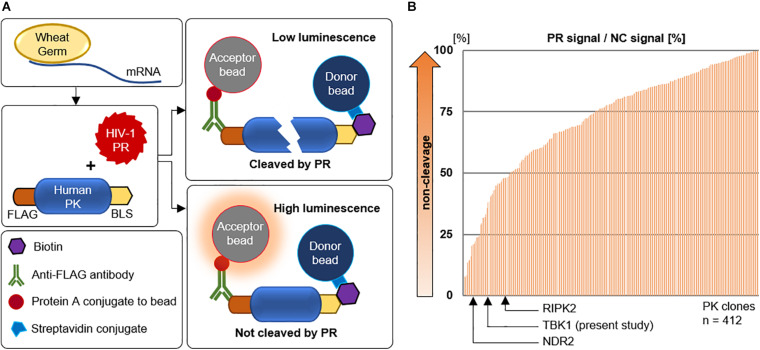
Screening for human kinases cleaved by HIV-1 protease. **(A)** Schematic representation of the AlphaScreen assay performed with HIV-1 protease and various human protein kinases generated by wheat germ cell-free protein synthesis. PR protease, PK protein kinase, GST glutathione S-transferase, BLS biotin linker sequence. **(B)** AlphaScreen assay result of the 412 human protein kinases arranged in order of cleavage to non-cleavage. TANK binding kinase-1 was identified in this study along with two other previously reported protein kinases. PR protease, NC negative control represents dihydrofolate reductase (DHFR), a protein that is not cleavable by HIV-1 protease.

In this context we intended to know if HIV-1 PR could interact and cleave other cellular protein kinases. We constructed a library of 412 human protein kinases and HIV-1 PR using the wheat germ cell free protein production system with the sequences listed in Supplementary Table 1. We then studied the interaction of HIV-1 PR with each of these kinases using the versatile AlphaScreen assay. The assay works on the principle of proximity-based luminescence which would be lost if the protein kinase is cleaved by HIV-1 PR (Figure 1A). Our screening assay detected the cleavage of previously reported PKs; receptor interacting protein kinases (RIPK), RIPK2 (Wagner et al., 2015), and Nuclear Dbf2-related (NDR) kinases, NDR2 (Devroe et al., 2005) in addition to several new targets (Figure 1B). Since these two PKs play direct or indirect roles in regulating innate immunity, we analyzed the other cleaved PKs that maybe involved in similar function. In this context, we selected TBK1 as it is an integral kinase in the IFN gene induction pathway, and we hypothesized that HIV-1 could cleave TBK1 to suppress type-I IFN production in order to evade innate immunity.

### HIV-1 Protease Cleaves TBK1

**FIGURE 2:**
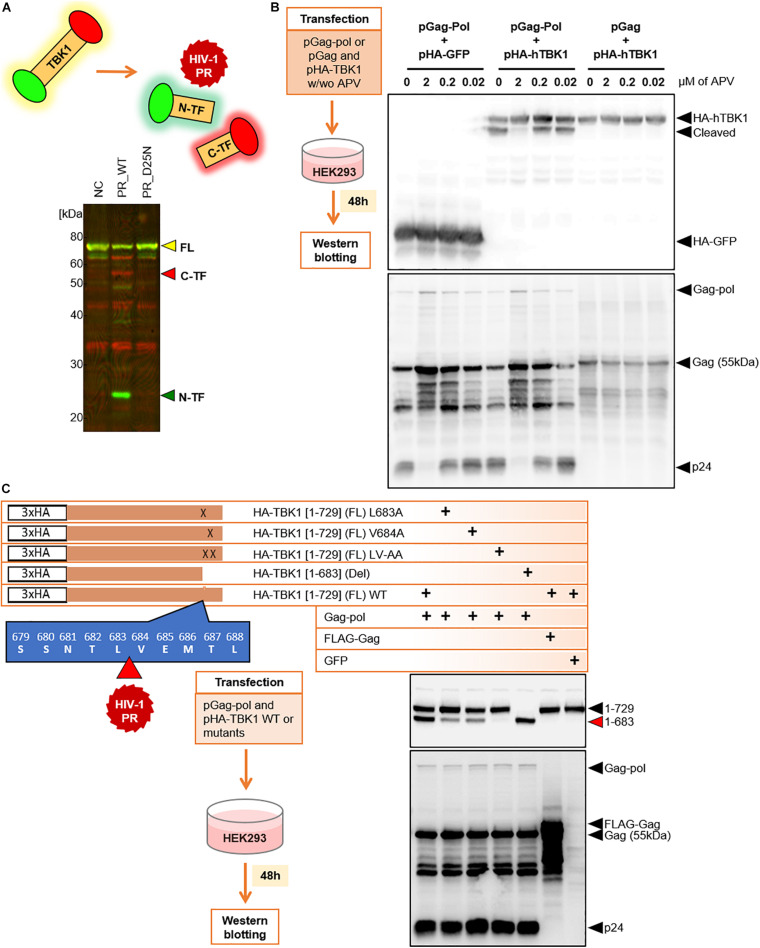
HIV-1 protease cleaves TBK-1. **(A)** Recombinant TBK1 protein with N-terminal green and C-terminal red fluorescence. Full length protein fluoresces yellow when intact. Upon *in vitro* proteolytic cleavage with HIV-1 PR, the N-terminal fragment fluoresces green while the C-terminal fragment fluoresces red detected by distinct bands in western blot. NC Negative control represents recombinant full length fluorescent TBK1 without addition of HIV-1 PR, FL Full length, C-TF C-terminal fragment, N-TF N-terminal fragment. **(B)** Western blot analysis 48 h after transfection of HEK293 cells with Gag-Pol or Gag with HA tagged TBK1 in the presence or absence of serial concentrations of amprenavir. Cleaved and non-cleaved TBK1 bands on the immunoblot were observed with anti-HA antibody. APV amprenavir. **(C)** Full length wild type TBK1, three cleavage site specific substitution mutants and one deletion mutant were generated with HA tag. Western blot analysis done 48 h after transfection of HEK293 cells with either of these TBK1 and pGag-Pol. TBK1 cleavage bands were observed with anti-HA antibody and p55 cleavage bands were visualized with anti-p24 antibody.

To visually observe the cleavage of TBK1 by HIV-1 PR in the extracellular milieu, we synthesized TBK1 with N-terminal Green (FLAG) and C-terminal red (biotin) tags by wheat cell-free system. Intact TBK1 appeared yellow due to the interference of the colors at either end of the protein. Two-color immunoblot analysis revealed that HIV-1 PR, but not its catalytic inactive mutant PR D25N, could cleave TBK1 protein into C-terminal red and N-terminal green fragments (Figure 2A). We then wanted to observe if HIV-1 PR mediated cleavage of TBK1 can occur intracellularly. HIV-1 Gag-Pol (enzymatically active) or Gag (devoid of enzymatic action) was co-transfected with TBK1 into HEK293 cells in the presence or absence of the protease inhibitor (PI) amprenavir. TBK1 was cleaved by Gag-Pol and not Gag, which was inhibited by amprenavir suggesting that HIV-1 PR exerts its proteolytic effect on TBK1 intracellularly (Figure 2B).

We further wanted to identify the site at which HIV-1 PR cleaved TBK1. For this, we harvested the TBK1 cleavage bands of proteins obtained upon *in vitro* HIV-1 PR treatment and outsourced them for amino acid sequencing. Sequencing results indicated that full length TBK1 (FL) was 729 amino acids (aa) long and HIV-1 PR cleaved TBK1 between L683 and V684 (direct data provided by the outsourced laboratory) (Figure 2C). Based on the sequencing data, we generated different types of TBK1 mutants; three cleavage site specific substitution mutants and one deletion mutant representing the cleaved TBK1 fragment (1–683) (Figure 2C). TBK1 wild type (WT) or the mutants were co-transfected with Gag-pol and then western blot was performed to observe the cleaved fragment. Gag-pol cleaved WT TBK1 which was denoted by the presence of a prominent cleavage fragment band. The substitution mutants were not cleaved effectively, and the deletion mutant was not cleaved (Figure 2C).

### Cleaved TBK1 Does Not Activate IFNβ Promoter

**FIGURE 3:**
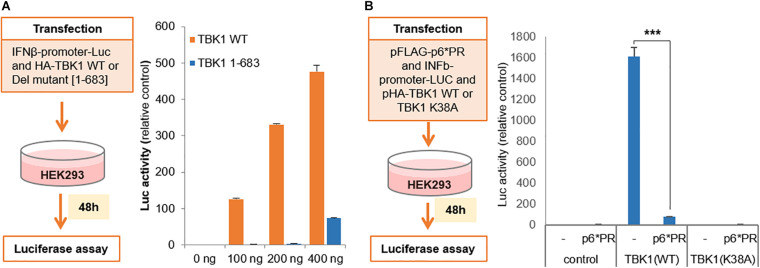
Cleaved TBK-1 does not activate IFNβ promoter. **(A)** Interferon β-promoter-Luc genetic element was co-transfected with serial concentrations of HA tagged wild type TBK1 or its deletion mutant [1–683] in HE293 cells. Relative luciferase activity denoting the interferon β promoter activity was assessed after 48 h of transfection. Bars represent the mean value of three replicates tested. **(B)** pFLAG-p6*PR, interferon β-promoter-Luc and either pHA-TBK1 wild type or non-functional mutant TBK1 K38A were co-transfected in HEK293 cells. Relative luciferase activity denoting the interferon β promoter activity was assessed after 48 h of transfection. Bars represent the mean value of three replicates tested. *** indicates *p*-value < 0.001.

We further wanted to check if the cleaved TBK1 is indeed incapable of activating type I IFN gene induction pathway. For this, we co-transfected HEK293 cells with IFNβ promoter tagged with secreted nanoluciferase (IFNβ-Luc) and full length TBK1 (1–729) or the cleaved TBK1 fragment (1–683) in increasing quantities and checked for luciferase activity. We could observe a dose dependent increase in the luciferase activity in with the full length TBK1 and not the cleaved fragment (Figure 3A), suggesting that the latter fails to activate the IFNβ promoter and thereby does not secrete IFNβ. This was further confirmed by triple co-transfection of HEK293 cells with IFNβ-Luc, HIV-1 PR and wild type TBK1 or functionally inactive TBK1 substitution mutant (TBK1 K38A). The wild type TBK1 accentuated the IFNβ promoter activity which was significantly inhibited in the presence of HIV-1 PR (Figure 3B).

### HIV-1 Protease Inhibits IRF3 Phosphorylation and Regulates Its Localization

**FIGURE 4:**
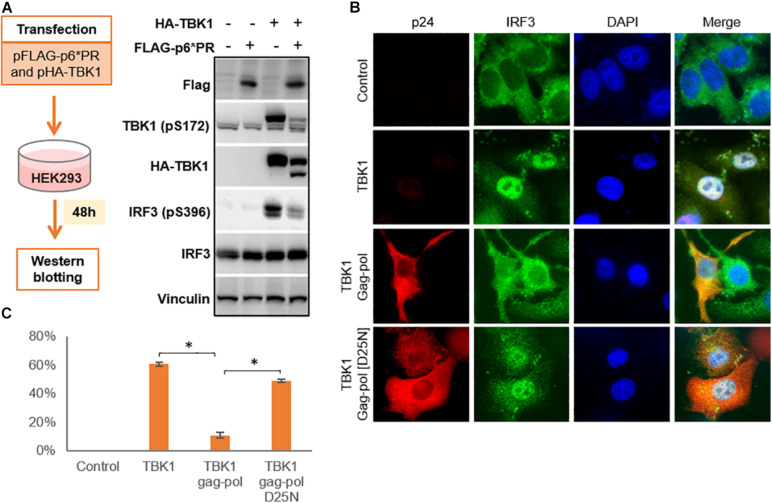
HIV-1 protease inhibits IRF3 phosphorylation and regulates its localization. **(A)** pFLAG-p6*PR and pHA-TBK1 were co-transfected into HEK293 cells and western blot was done after 48 h. Total TBK1, phosphorylated TBK1, total IRF3 and phosphorylated IRF3 were observed with corresponding antibodies. **(B)** Cells co-transfected with TBK1 and wild type Gag-pol or its enzymatically inactive mutant D25N for 48 h after which cells were collected and fixed in paraformaldehyde. Immunofluorescence staining done for gag-pol with AlexaFluor 568 and IRF3 stained with AlexaFluor 488 which emitted red and green fluorescence, respectively. Nucleus stained blue with DAPI. Control represents non-transfected cells. **(C)** Percentage of IRF3 activation was calculated from the IRF3 panel of Figure 4B depicting the number of cells containing IRF3 translocated into the nucleus over total cells in the field. Data represents the mean value obtained from counting three fields containing at least 20 cells in each field. ^∗^ indicates *p*-value < 0.05.

TBK1 undergoes autophosphorylation in order to phosphorylate cytoplasmic IRF3 which then translocates to the nucleus to activate transcription that leads to IFN production. We transfected HIV-1 PR and TBK1 into HEK293 cells and harvested the cells after 48 h to perform a western blot in order to observe the role of HIV-1 PR in the phosphorylation of TBK1 and IRF3 (Figure 4A). In the absence of HIV-1 PR, we observed prominent bands of both phosphorylated TBK1 (pS172) and IRF3 (pS396). However, both bands were faint in the presence of HIV-1 PR suggesting that TBK1 cleaved by HIV-1 PR does not get phosphorylated and hence does not phosphorylate IRF3.

We then performed an immunofluorescence study to observe the localization of IRF3 in the presence or absence of HIV-1 PR. Cells co-transfected with TBK1 alone or along with either wild type gag-pol (WT) or catalytically inactive mutant Gag-pol (D25N). WT gag-pol cleaved TBK1 so that IRF3 was not phosphorylated and hence remained in the cytoplasm. Whereas TBK1 was not cleaved by gag-pol D25N, so IRF3 was phosphorylated and translocated into the nucleus in a similar pattern to that seen with TBK1 alone (Figures 4B,C).

### Drug Resistant Mutant Proteases Have Diminished Efficacy in Cleaving TBK1

**FIGURE 5:**
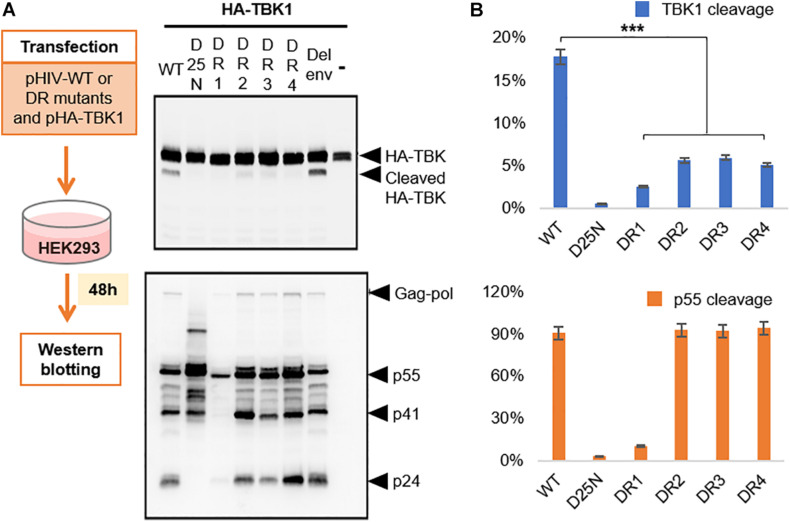
Drug resistant proteases cleave TBK1 ineffectively. **(A)** Wild type HIV or either of the four drug resistant mutants listed in Table 1 were co-transfected with HA tagged TBK1. Western blot analysis done after 48 h to observe TBK1 cleavage bands with anti-HA antibody and p55 cleavage bands with anti-p24 antibody. **(B)** Quantification of band intensities from **(A)**. Graph on the top indicates cleavage ratio percentage of cleaved TBK1 over un-cleaved TBK1 while the one on the bottom indicates that of p24 over p55. *** indicates *p*-value < 0.001.

The widespread use of protease inhibitors as principal agents for anti-retroviral therapy (ART) has selected out several HIV-1 PR mutants which show cross resistance to several PIs (Rhee et al., 2010). However, the mutations in PR gene are often known to compromise the pathogenic fitness of HIV-1 (de Vera et al., 2013). The principal substrate of HIV-1 PR is the viral precursor polyprotein which it cleaves within the virion to enable maturation (Yang et al., 2012). So, we were intrigued to know if these drug resistance mutations could differentially affect the ability of HIV-1 PR to cleave TBK1 and viral precursor protein. We selected four HIV-1 PR mutants DR1-4 (Table 1) to study their ability to cleave TBK1. HIV-1 PR WT or the catalytically inactive mutant D25N or either of the drug resistant mutants DR1-4 were co-transfected along with TBK1 in HEK293 cells and western blot was done after 48 h to check for the presence of TBK1 cleavage fragment band. While the HIV-1 PR WT produced a prominent cleavage band, none of the four drug resistant mutants and D25N cleaved TBK1 effectively (Figures 5A,B). Interestingly, the p55 precursor protein was cleaved into p41 and p24 by all the mutants except the replication defective DR1. This finding suggests that mutations in HIV-1 PR alter the enzyme’s substrate specificity. Mutant PRs retain their ability to cleave polyproteins into their functional sub-units while losing their ability to cleave TBK1.

**TABLE 1:** HIV-1 protease mutants resistant to protease inhibitors.

**Clone**	**Major resistance mutations**	**Minor resistance mutations**	**Other mutations**
DR1	N88S	L10F, T74S, L89V	I13V, K20T, E35D, M36I, R41K, I62V, L63P, H69K
DR2	N88S	L10F, A71T, L89V	I13V, K20T, E35D, M36I, R41K, I62V, L63P, H69K
DR3	N88S	L10F, A71T, L89V	I13V, K20T, E35D, M36I, R41K, L63P, H69K
DR4	G48S, I54V, V82F, L90M	L10I	I13V, G16Q, K20I, E35N, M36I, P39Q, R41K, K45V, I62V, L63P, H69K, L89M

### Protease Inhibitors Rescue IFN-I in Infected Cells

**FIGURE 6:**
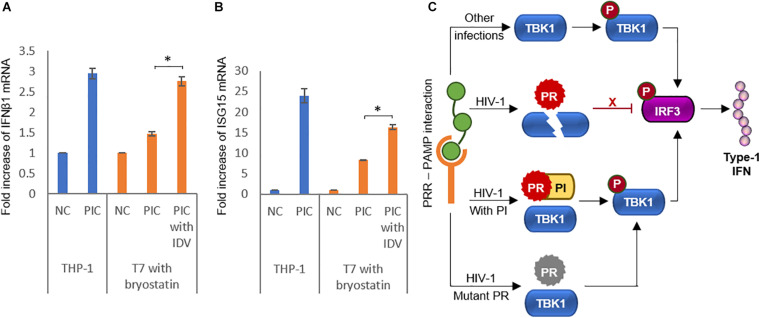
Protease inhibitors rescue interferon activity in infected cells. **(A)** HIV-1 latently infected monocytic cell line T7 was reactivated with 25 ng/ml bryostatin-1 and IFNβ1 mRNA was detected by RT-qPCR in poly(I:C) treated cells in the presence or absence of the protease inhibitor indinavir. Uninfected THP-1 cells were used as positive control. Bars represent the mean value of three replicates. NC Negative control, PIC Poly(I:C), IDV Indinavir. ^∗^ indicates *p*-value < 0.05. **(B)** HIV-1 latently infected monocytic cell line T7 was reactivated with 25 ng/ml bryostatin-1 and ISG15 mRNA was detected by RT-PCR in poly(I:C) treated cells in the presence or absence of the protease inhibitor indinavir. Uninfected THP-1 cells were used as positive control. Bars represent the mean value of three replicates. NC Negative control, PIC Poly(I:C), IDV Indinavir. ^∗^ indicates *p*-value < 0.05. **(C)** Schematic representation of the findings of the study summarizing the role of HIV-1 protease in the interferon secretion pathway.

As we had observed a clear and present effect of HIV-1 PR on TBK1 and its reversal by PIs in transfection studies, we wanted to find if these effects were observable in HIV-1 infected cell lines. We used the uninfected monocytic cell line THP1 as comparison and studied the effects of PIs in the HIV-1 latent infected cell line T7. We transfected the cells with poly(I:C), a robust inducer of IFN-1 to induce its production in both cell types (Ranganath et al., 2016). To activate HIV-1 PR production in T7 cells, we treated them with bryostatin-1, a potent HIV-1 latency reactivating agent known for its superior *in vitro* action on monocytic cell lines (Martínez-Bonet et al., 2015). We observed an increase in IFNβ1 mRNA production in reactivated T7 cells treated with PIs suggesting that PIs relieved the inhibitory effect of HIV-1 PR on IFN-1 secretion pathway (Figure 6A). We further wanted to check if this increase in IFNβ1 mRNA caused by PIs translated to downstream effects. The interferon-stimulated gene ISG-15 mRNA was found to be upregulated in PI treated cells, suggesting the effect of IFNβ1 rescued by PIs (Figure 6B).

## Discussion

PR is an important enzyme of HIV-1 coded by the pol gene along with the other enzymes, reverse transcriptase and integrase. Unlike the other two which are required for viral replication in the intracellular milieu, PR has long been considered as a late phase enzyme known to act inside the newly budded virions where it cleaves the viral precursor polyproteins into functional subunits to produce mature infectious progeny (Konvalinka et al., 2015). However, there is accumulating evidence that HIV-1 PR plays an accessory role to interfere with host cellular functions by cleaving various intracellular proteins (Wagner et al., 2015). One of the earliest observations in this regard is the induction of apoptosis in infected cells by HIV-1 PR (Ventoso et al., 2005). Although the exact mechanism is unknown, it is presumed to be due to cleavage of different host proteins related to the apoptotic pathway such as Bcl-2 (Strack et al., 1996) and Procaspase 8 (Sainski et al., 2011). Although this action of HIV-1 PR is attributed to the reduction of CD4 T-cells during productive infection and in terminal disease, HIV-1 PR mediated apoptosis makes it deleterious for the virus to replicate.

More recent studies have revealed the role of HIV-1 PR in cleaving the host cellular proteins involved in inflammation and immune response in order to dampen the host defenses and make the intracellular environment conducive for viral replication. The salient observation in this regard is the proteolytic cleavage of host PKs involved in innate immunity by HIV-1 PR. Nuclear Dbf2-related (NDR) kinases, NDR1 and NDR2 are essential PKs instrumental in regulating PRR and cytokines in innate immunity (Ye et al., 2020) and HIV-PR has been shown to cleave both NDR1 and NDR2 (Devroe et al., 2005). Similarly, receptor interacting protein kinases (RIPK) RIPK1 and RIPK3 are involved in eliminating virus infected cells through necroptosis and have also been shown to mediate type 1 IFN signaling for this purpose (Rajput et al., 2011). RIPK1 has been reported to be cleaved by HIV-1 PR during infection (Wagner et al., 2015). In similar lines, our study shows that TBK1 is yet another host PK that is cleaved by HIV-1 PR to inhibit type 1 IFN production possibly to dampen innate immunity and promote viral infection.

Different accessory proteins of HIV-1 have been identified to directly interfere in the IFN-I secretory pathway in different host cells to suppress IFN-I production and the mechanisms vary in productive and latent infections. These viral proteins are expressed in productively infected T-cells where they reduce IFN-I production by degrading IRF3. The proteins Vpr and Vif mediate ubiquitination of IRF3 and cause its subsequent destruction in proteasomes (Okumura et al., 2008) while Vpu interacts with IRF3 to redirect the latter into lysosomes for proteolytic degradation (Doehle et al., 2012). In productively infected dendritic cells and macrophages, Vpr and Vif physically interact with TBK1 to prevent its autophosphorylation thereby inhibiting the subsequent steps of IFN-I production (Harman et al., 2015).

Like productively infected cells, IFN-I production is impaired in cells that are latently infected with HIV-1, but the mechanisms involved in the latter are not as clearly defined as in the former (Ranganath et al., 2016). As expression of accessory proteins are unlikely in latent infection, there must be other mechanisms by which HIV-1 diminishes IFN-I production in these cells. Wagner et al. (2015) have demonstrated that PKs are cleaved by HIV-1 PR expressed by proviral genes after integration and not by the PRs brought in by the incoming viruses during early infection. This could possibly explain that non-PR mediated mechanisms are responsible for IFN suppression in acute productive infection while PR plays a role in IFN suppression in latently infected cells. Rigogliuso et al. (2019) have demonstrated the expression of PR in cells containing proviral genomes of Human endogenous retroviruses (HERVs) and its ability to cleave several cellular proteins. Hence it is interesting to speculate if HIV-1 PR is subtly expressed in latently infected cells to suppress IFN-I production, warranting the need for further studies to identify this phenomenon.

Another interesting observation in western blots was the presence of considerable amount of non-cleaved full length TBK1 bands in addition to the cleavage band (Figures 2B, 4A) denoting that HIV-1 PR cleaves only a fraction of the expressed TBK1. It is intriguing why this non-cleaved TBK1 did not participate in further downstream events to secrete IFN-I despite being present in more copious amounts than their cleaved counterpart. Further studies are needed to detect whether the cleaved fragments possibly hinder the function of the non-cleaved TBK1.

Protease inhibitors are highly effective drugs against HIV-1 and comprise the mainstay agents of ART. With our *in vitro* experiments it is evident that PIs can prevent HIV-1 from cleaving TBK1, suggesting that PIs could improve the innate immune response to facilitate viral clearance. Harman et al. (2015) have shown the effect of HIV-1 PR in cleaving host cellular proteases in an infection model comprising previously uninfected primary CD4 T-cells. We have shown a similar effect of HIV-1 PR in latently infected monocytic cell lines upon reactivation. However, the possibility of this happening *in vivo* remains to be studied.

Due to their widespread use, PIs have selected drug resistant mutants. It has been observed that mutations of protease that enable the enzyme to become resistant to PIs paradoxically reduce the pathogenic fitness of the virus (de Vera et al., 2013). We were able to demonstrate that mutations in PR which confer resistance to PIs make the enzyme less potent to cleave TBK1 while still retaining its ability to cleave the viral polyprotein into functional units to cause viral maturation. Except for one mutant (DR1) which was proliferation defective, all the other three mutants effectively cleaved gag-pol but showed inefficiency to cleave TBK1. This suggest that in order to retain its basic biological function, the evolved virus must pay the price of becoming more vulnerable to innate immune clearance.

Achieving a functional cure to HIV infection depends on successfully eradicating the replication competent viruses from the latent reservoir. However, all attempts till date to shock and kill the proviruses have failed miserably. Palermo et al. (2019) have demonstrated that latency reactivation strategies work better when there is innate immune activation in addition to epigenetic modulation rather than the latter alone. Since HIV-1 uses multiple mechanisms to evade innate immunity, it could be a possible reason for the inefficiency of the shock and kill strategy. We have unraveled a novel mechanism where HIV-1 PR is involved in degradation of TBK1, one of the major proteins responsible in IFN-I secretion. This action could be counteracted by PIs and the drug resistance mutations in PR reduce the enzyme’s efficiency to degrade TBK1 (Figure 6C). We suggest that the molecular link between HIV-1 PR and IFN-I secretion could play an important role in the pathophysiology of HIV-1 and highlight the need to study this in further detail in future studies.

## Data Availability Statement

The datasets presented in this study can be found in online repositories. The names of the repository/repositories and accession number(s) can be found in the article/Supplementary Material.

## Author Contributions

SJ, AK, KM, SM, and MN performed most of the experiments. AK, AT, TS, and AR analyzed the data. SJ, KM, and SM designed the experiments and wrote the manuscript. AT, TS, and AR revised and edited the manuscript. AR conceived the idea and supervised the project. All authors made significant contributions to the article and approved the submitted version.

## Conflict of Interest

The authors declare that the research was conducted in the absence of any commercial or financial relationships that could be construed as a potential conflict of interest.
